# Effect of Boronizing on the Microstructure and Mechanical Properties of CoCrFeNiMn High-Entropy Alloy

**DOI:** 10.3390/ma16103754

**Published:** 2023-05-16

**Authors:** Mingyu Hu, Xuemei Ouyang, Fucheng Yin, Xu Zhao, Zuchuan Zhang, Xinming Wang

**Affiliations:** 1 School of Materials Science and Engineering, Xiangtan University, Xiangtan 411105, China; 202021001605@smail.xtu.edu.cn (M.H.); fuchengyin@xtu.edu.cn (F.Y.); 202121551594@smail.xtu.edu.cn (X.Z.); 202005720811@smail.xtu.edu.cn (Z.Z.); wangxm@xtu.edu.cn (X.W.); 2 Key Laboratory of Materials Design and Preparation Technology of Hunan Province, Xiangtan University, Xiangtan 411105, China

**Keywords:** high entropy alloy, boronizing, growth kinetics, boronizing mechanism, wear resistance

## Abstract

The CoCrFeNiMn high-entropy alloys were treated by powder-pack boriding to improve their surface hardness and wear resistance. The variation of boriding layer thickness with time and temperature was studied. Then, the frequency factor D_0_ and diffusion activation energy Q of element B in HEA are calculated to be 9.15 × 10^−5^ m^2^/s and 206.93 kJ/mol, respectively. The diffusion behavior of elements in the boronizing process was investigated and shows that the boride layer forms with the metal atoms diffusing outward and the diffusion layer forms with the B atoms diffusing inward by the Pt-labeling method. In addition, the surface microhardness of CoCrFeNiMn HEA was significantly improved to 23.8 ± 1.4 Gpa, and the friction coefficient was reduced from 0.86 to 0.48~0.61.

## 1. Introduction

The equiatomic CoCrFeNiMn alloy with an fcc structure shows high ductility and high fracture toughness even at significantly lower temperatures [1,2,3]. However, more slip systems in fcc solid solution [4,5,6] caused lower hardness of CoCrFeNiMn [7,8] and worse wear resistance [9]. Alloying is a crucial method to improve strength and wear resistance. Some expensive and refractory alloying elements such as W, Ta, Nb, and Mo are added to CoCrFeNiMn HEA. This method, however, reduces its plasticity [10,11,12,13]. Thus, preparing a reinforced layer on the surface of CoCrFeNiMn HEA is a vital method to solve this problem. In many applications, the service life of materials depends highly on their surface properties, which can be extended by improving the wear resistance of the surface. Thus, preparing a reinforced layer on the surface of CoCrFeNiMn HEA is a vital method to solve this problem.

Boronizing is a thermo-chemical surface treatment method used to enhance the surface hardness and wear resistance of ferrous materials [14,15,16,17]. This method is used to enhance the surface properties of ferrous materials, while the boriding behavior of various steels has been extensively studied. The boride layer (BL) formed on the steel surface by boriding treatment can significantly improve its surface properties [18,19,20]. The principal elements in CoCrFeNiMn HEA, such as Fe, Co, and Cr, have a strong tendency to form stable borides [18,21]. It is reported that the wear resistance of boronized samples exceeds two times the wear resistance value of carburized, carbo-nitrided, or chromium-plated samples [22,23]. Hou et al. [24] obtained the boronized layer on the Al_0.25_CoCrFeNi HEA by the solid-boronizing method. A boronized layer with the phases (Ni, Co, Fe)_2_B and CrB has been prepared for holding for 9 h at 900 °C. The microhardness of the boronized sample surface approximately approaches 1136 HV_5_, which is six times that of the untreated HEA. Hiroaki Nakajo et al. [25] obtained ceramic layers on the surface of CoCrFeMnNi HEA using the SPS method, and a powdered mixture of B_4_C and KBF_4_ was used as the boron source. M_2_B, MB, and Mn_3_B_4_ type borides were formed on the surface of HEA, and the surface hardness reached 2000~2500 Hv_0.1_. Most studies are focused on the microstructure and mechanical properties of borided bulk HEAs after boriding, but there are few reports on the boronizing mechanism of HEA [26,27].

In this study, a reinforced layer was prepared on the surface of HEA by powder-pack boriding and focused on the effect of temperature and duration on the boronizing of CoCrFeNiMn HEA. The boronized samples were characterized using various methods. Furthermore, the properties of boronized CoCrFeNiMn alloys and diffusion behavior were investigated. This study could provide a reference for high hardness and high wear resistance HEA that could be used in a variety of industrial applications, such as the mold and crucible used in the cast aluminum industry [28,29], because the borides formed on the surface have high hardness, excellent corrosion resistance, and wear resistance [30,31], which allow HEA to be applied in special environments to increase service life.

## 2. Materials and Methods

### 2.1. Alloys Preparation

Alloy ingots with a nominal composition of Co_20_Cr_20_Fe_20_Ni_20_Mn_20_ (in at.%) were fabricated by the vacuum arc-melting method using a non-consumable tungsten electrode. High-purity metals were used and melted at least three times to promote chemical homogeneity. The as-cast alloy was homogenized at 1200 °C for 24 h under a vacuum environment and then cut into 6 × 6 × 3 mm and polished with 200#, 400#, 600#, and 800# sandpaper and diamond solution. Additionally, a Pt-labeled layer was deposited on the surface of the annealed sample using an auto-fine coater (JEOL JFC-1600) with a sputtering time of 300 s to determine the evolution of the diffusion of the elements during boriding.

### 2.2. Boronizing Process

Boronizing treatment was performed at 850, 900, and 950 °C for 3 h, 6 h, 9 h, 12 h, 15 h, and 18 h, respectively. The annealed CoCrFeNiMn samples were placed in the alumina crucible, then packed with high-purity boron powder. The alumina crucibles were tightly closed by a high-temperature sodium silicate and kaolin binder to make them airtight. After boriding was completed, the sealed crucible was extracted and air-cooled to room temperature, and the samples were ultrasonically cleaned using anhydrous ethanol to remove the boron powder from the surface.

### 2.3. Characterization

The cross-sectional microstructures were observed using a scanning electron microscope (SEM, EVO MA10) equipped with energy dispersive spectrometry (EDS). However, it was difficult to quantify the B atom by EDS, so the element composition of BL was tested on the electron probe microanalysis (EPMA, Shimadzu 1720, beam spot diameter 1 μm, acquisition time 10 s) equipped with a wavelength dispersive spectrometer (WDS). The phase structure was characterized by X-ray diffraction (XRD, Rigaku Ultimate IV) with Cu-Ka radiation. The friction and wear measurements were performed using a ball-on-plate tribometer (CFT-I Tribometer) under dry sliding conditions at 25 °C in an open-air atmosphere. The hardness and Young’s modulus of the BL were measured using a nano-indenter.

## 3. Results

### 3.1. Microstructure Analyses

**Figure 1:**
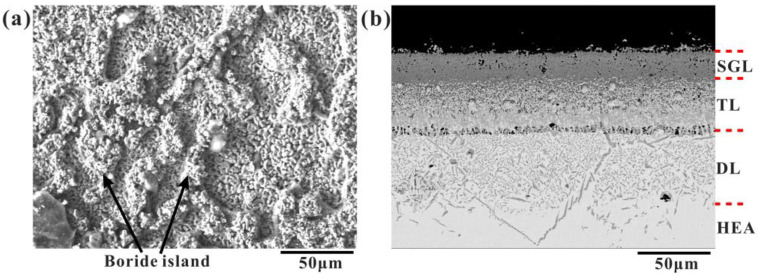
SEM images of boronized CoCrFeNiMn HEA at 950 °C for 9 h: (**a**) surface, (**b**) cross-section.

Figure 1 shows the surface and cross-sectional morphological images of the CoCrFeNiMn high-entropy alloy after boron penetration at 950 °C for 9 h. The results show that the surface of the high-entropy alloy after boriding is formed by many round rod-like boride assemblies, and there are discontinuous islands of borides, as shown in Figure 1a. According to the different contrast and phase numbers, the surface layer of boronized HEA can be divided into a single-phase layer (SGL), a transition layer (TL), a diffusion layer (DL), and a HEA substrate [32].

**Figure 2:**
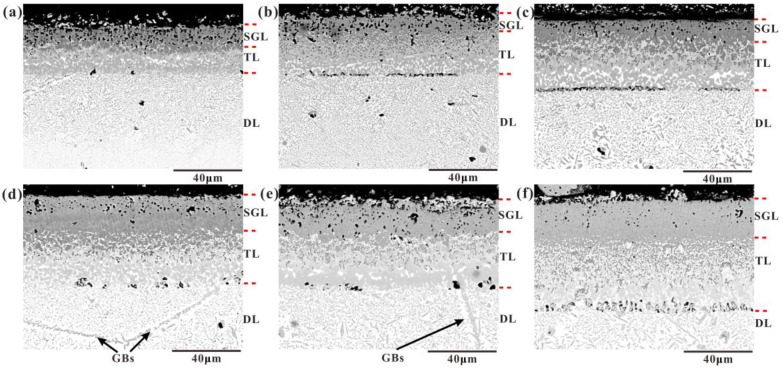
Cross-sectional SEM images of the CoCrFeNiMn HEA substrate with the boronizing times of (**a**) 3 h, (**b**) 6 h, (**c**) 9 h, (**d**) 12 h, (**e**) 15 h, and (**f**) 18 h.

Figure 2 shows the cross-sectional SEM images of the CoCrFeNiMn HEA boronized at 950 °C at different times. As can be seen, the surface layer of the samples at different times is composed of the BL, the diffusion layer, and the substrate. The interface between the BL and the DL was smooth, which differs from the saw-tooth shape for the boronized pure iron and iron alloys. While the BL consists of an SGL and a two-phase TL, some discontinuous pores were also observed on the interface and the SGL; the interface between them is a saw-tooth shape. The thickness of the BL and total layer were measured, and the average thickness of the BL was 31.58, 41.7, 47.51, 51.09, 53.73, and 64.35 µm for the boronized alloys for 3 h, 6 h, 9 h, 12 h, 15 h, and 18 h, respectively.

### 3.2. Growth Kinetics Analyses

**Figure 3:**
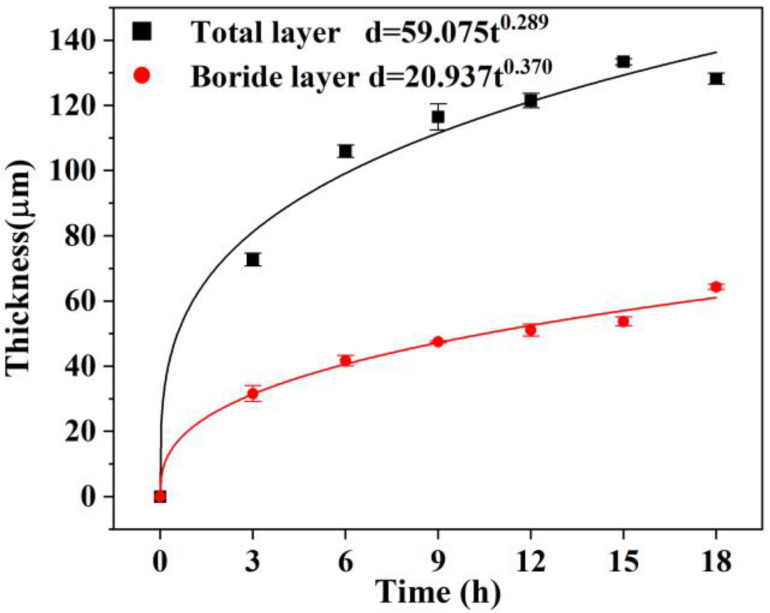
Variation of the boride layer thickness as a function of time for the boronized HEAs.

Previous studies have mainly investigated the growth kinetics of steel and its alloys. Andrijana Milinović et al. [33] investigated the growth kinetics of different steels, and the results showed that the frequency factor D_0_ and diffusion activation energy Q of elemental boron in C15 steel are 3.17 × 10^−4^ m^2^/s and 194.80 kJ/mol, respectively. Figure 3 shows the BL and the whole layer thickness as a function of time. It can be noticed that the thickness of the BL and the whole layer increases with the increase in boronizing time. With high-purity B powder as a boronizing agent, the influence of Si, C, and F in conventional boriding agents on the properties of BL can be excluded, which also simplifies the analysis of the diffusion behavior of different elements during the boronizing process. The growth rate was reduced significantly as the boronizing time increased.

The diffusion rate is commonly expressed by the formula shown in Equation (1) [34]:

d = At^n^ (1)

where d is the thickness of the BL, A is a time constant, t is the time, and n is the diffusion rate exponent. It is shown that the diffusion rate exponent n was calculated to be 0.370 by the BL thickness exponential fitting curve expression d = 20.937t^0.370^ and the fitting curve is closer to parabolic. According to Wagner’s hypotheses [35], this parabolic growth can imply that a diffusion process controls the boronizing rate.

The function between the thickness of the BL and time is in accordance with the parabolic law, and the expression is shown in Equation (2):

d^2^ = Dt (2)

where d is the thickness of the diffusion layer (m), D is the growth rate constant (m^2^/s), and t is the diffusion time (s). Since it is a quadratic equation, the solution is obtained by finding its roots, combined with the Arrhenius formula:

D = D_0_·e^−Q/(RT)^ (3)

where D_0_ is the frequency factor (m^2^/s), Q is the diffusion activation energy (kJ/mol), T is the diffusion temperature (K), and R is the universal gas constant (kJ/(mol·K)). The frequency factor indicates the rate of molecular collisions in the reaction.

(4) $$lnD=lnD_{0}-\frac{Q}{R}\cdot \frac{1}{T}$$

**Figure 4:**
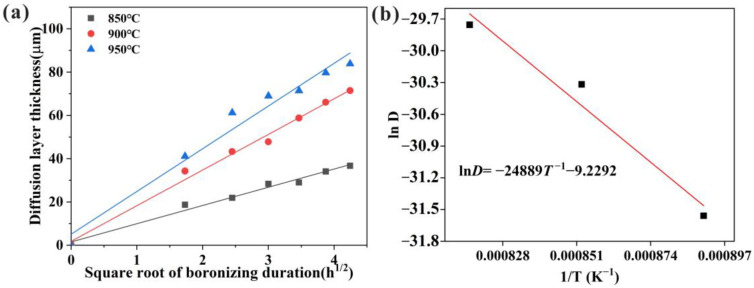
Growth kinetics of boride layers on HEA: (**a**) Boride layer thickness as a function of the square root of the boronizing duration; (**b**) natural logarithm of the growth rate constant as a function of the reciprocal boronizing temperature.

Figure 4a, plotted according to Equation (3), shows the linear relationship between the thickness of the diffusion layer and the square root of time at different temperatures. According to Equation (4), the relationship between the growth rate constant’s natural logarithm and the diffusion temperature’s reciprocal can be represented by a line with slope Q/R, and lnD_0_ is the intersection of the line with the vertical coordinate. The value of the growth rate constant was obtained from the slope of the line; the fitted curve of the natural logarithm of the growth rate constant and the inverse of the temperature showed a linear relationship, as shown in Figure 4. The value of the diffusion activation energy of B in the CoCrFeNiMn HEA is determined by the slope of the line, while the natural logarithm of the frequency factor is determined by the intersection of the extrapolated line with the vertical coordinate. These values are given in Table 1, while the D_0_ of HEA is lower than the D_0_ of C15 due to the sluggish diffusion effect of HEA.

### 3.3. Phase Structure and Chemical Composition

**Figure 5:**
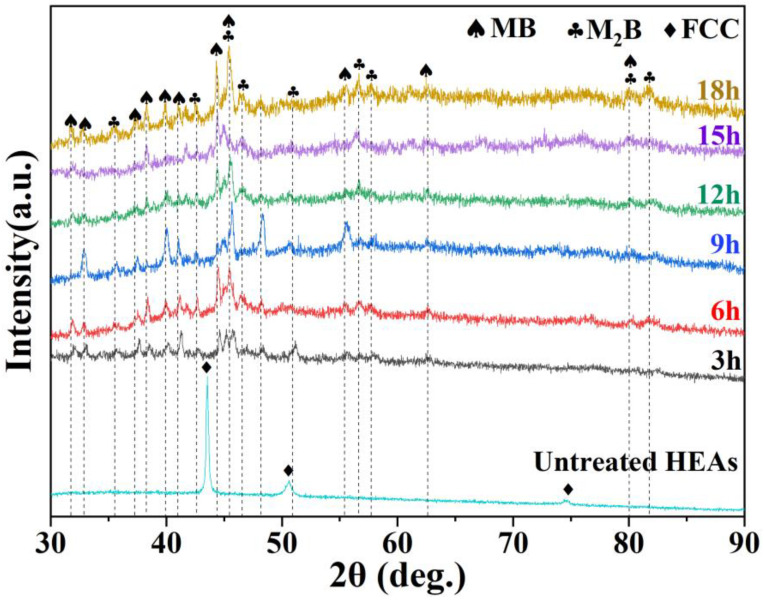
XRD patterns of boronized samples at 950 °C for different times.

An XRD analysis was performed on the surface of the boronized HEA, as shown in Figure 5. The main diffraction peaks appear in the same position for boronized samples, and the BL consists of MB (M = Co, Cr, Fe, Ni, and Mn) and M_2_B phases. While MB and M_2_B are formed due to the replacement of Fe atoms in FeB and Fe_2_B by Co, Cr, Ni, and Mn.

**Figure 6:**
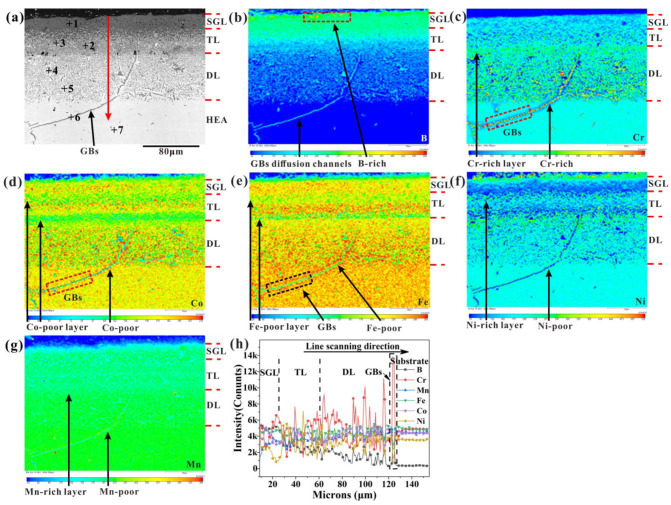
(**a**) Cross-sectional SEM images of boronized CoCrFeNiMn HEA at 950 °C for 9 h, (**b**–**g**) EPMA elemental mapping of B, Cr, Co, Fe, Ni, and Mn, respectively, (**h**) EPMA line analysis.

The composition of each phase in boronized HEA from surface to substrate was tested by WDS, as shown in Figure 6a. The composition of the seven phases is listed in Table 2. The BL has three different phases: the dark gray phase (spot 1), the gray phase (spot 3), and the white phase (spot 2). The element composition of BL was tested on the EPMA equipped with a WDS. The concentrations of B and M (M = Co, Cr, Fe, Ni, and Mn) at the outer layer are 45.49 at.% and 54.51 at.%, respectively. The atomic ratio B:M is close to 1:1. In addition, the XRD patterns of the boride surface show the existence of the MB, which has a similar microstructure to the CrB. Therefore, the single phase is identified as MB-type boride. According to the results of points 2–7, the phases from the surface to the substrate can be determined to be single-phase MB, Cr-rich MB + Ni-rich MB, Cr-rich M_2_B + Ni-rich M_2_B, Cr-poor FCC + Cr-rich M_2_B, and FCC substrate. The EPMA elemental mapping and line analysis of the boronized CoCrFeNiMn HEA for 9 h are shown in Figure 6b–h. The concentration of B increases from surface to substrate, as shown in Figure 6b. B atoms mainly exist in the boride, and their solubility in an fcc solid solution is minimal. Based on the distribution of the Cr element, as shown in Figure 6c, Cr atoms have obvious segregation and are mainly concentrated in the DL and grain boundaries in the substrate close to the DL. The Cr element preferentially combines with the B element to form a Cr-rich boride. The distribution of Co, Fe, Ni, and Mn elements shows obvious delamination in the BL, and the (Co, Fe, Ni, Mn)-poor layer and the (Co, Fe, Ni, Mn)-rich layer are alternately arranged as shown in Figure 6d–g. Moreover, the content of these elements at the grain boundary was significantly lower than that in the grain. Figure 6h shows the line analysis from surface to substrate marked with arrows in Figure 6a, which further indicates the existence of the element segregation phenomenon.

### 3.4. Boronizing Mechanism

**Figure 7:**
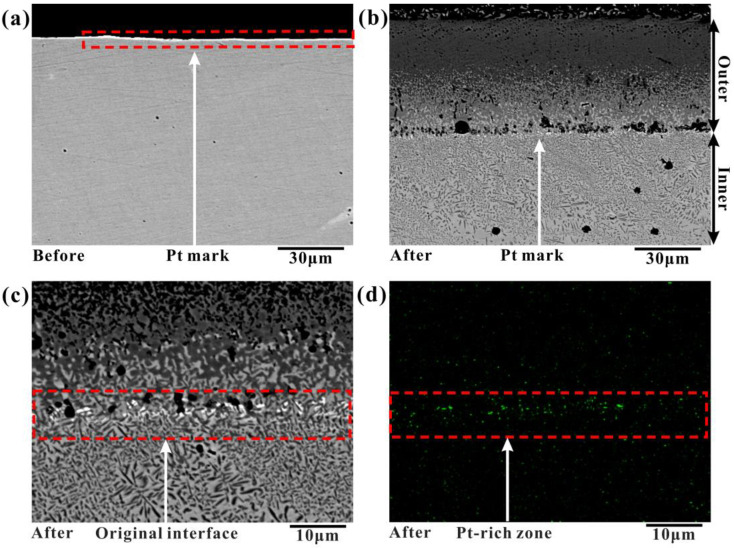
Cross-sectional SEM image of the Pt-marked CoCrFeNiMn HEA: (**a**) before boronizing, (**b**) after boronizing at 950 °C for 9 h, (**c**) the photograph of the original interface, (**d**) elemental mapping of Pt.

Previously, Cengiz et al. [36] investigated the boronizing mechanisms of CoCrFeNi alloy and CoCrFeNiTi alloy. The diffusion of B and Si elements mainly occurred in the boronizing process, and the diffusion behavior of metal elements was not considered in their work. To investigate the diffusion behavior of elements during boronizing, the boronizing experiments were carried out using the Pt element labeling method. This method has been used in the past to study the oxidation behavior of steel [34,37]. The Pt layer shown in Figure 7a was sprayed on the surface of the annealed CoCrFeNiMn alloy before boronizing. Boronizing was performed at 950 °C for 9 h. The cross-sectional morphology of the boronized samples was obtained, as shown in Figure 7b,c. Comparing the interface morphology between the Pt-sprayed and unsprayed samples, it was observed that there was almost no difference between the samples except for the quantity and size of pores at the interface between the BL and the diffusion layer, indicating that the effect of the markers on boronizing was minimal. The Pt element is distributed between the boride and diffusion layers, and no Pt element is found in other locations, as shown in Figure 7d. It indicates that Pt did not diffuse during boronizing and that the interface moves outward from the position of the Pt mark after boronizing. The Pt-labeled experiment confirms the proposed mechanism, such as the “available space model” [38,39,40,41,42,43,44] in oxidation, which is the outward migration of metal atoms forming the BL and the inward migration of B forming the diffusion layer.

**Figure 8:**
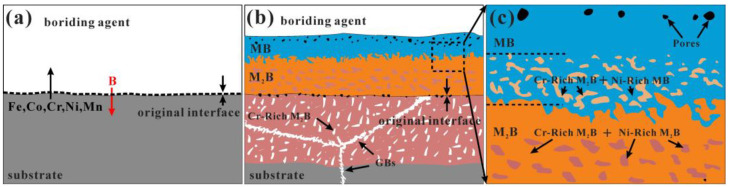
Schematic views of the boronizing mechanism of the CoCrFeNiMn HEA. (**a**) before boronizing, (**b**) after boronizing, (**c**) local magnification of (**b**).

The schematic model of the boronizing mechanism is suggested based on the results above, as shown in Figure 8. The B atoms can be diffused easily into the surface of alloys due to their relatively small size (0.087 nm), and their diffusion is very fast at high temperatures. In the early stage, B atoms preferentially diffuse to the substrate along the grain boundary and then react easily with alloying elements, forming Cr-rich boride, while metal atoms diffuse outward and form M_2_B-type boride. Meanwhile, due to the mutual diffusion of the metal atoms and B atoms, vacancies accumulate at the metal/boride interface, and metal creep cannot compensate for the volume of metal consumed by diffusion to the outer layers, resulting in the formation of pores at the metal/boride interface. The EPMA point analysis and WDS mapping results showed the existence of the element segregation phenomenon, and the content of Cr and Ni elements in boride changed most obviously. With increasing boronizing time, the thickness of the BL and DL increases, forming the microstructure as shown in Figure 8b. In addition, the metal atoms that diffuse to the surface are relatively smaller than those on the inner side and are unable to fully combine with the boriding agent to form borides. The retained B powder is removed from the SGL after grinding and polishing the samples, leaving the pores on the SGL, as shown in Figure 8c.

### 3.5. Hardness and Wear Behavior

**Figure 9:**
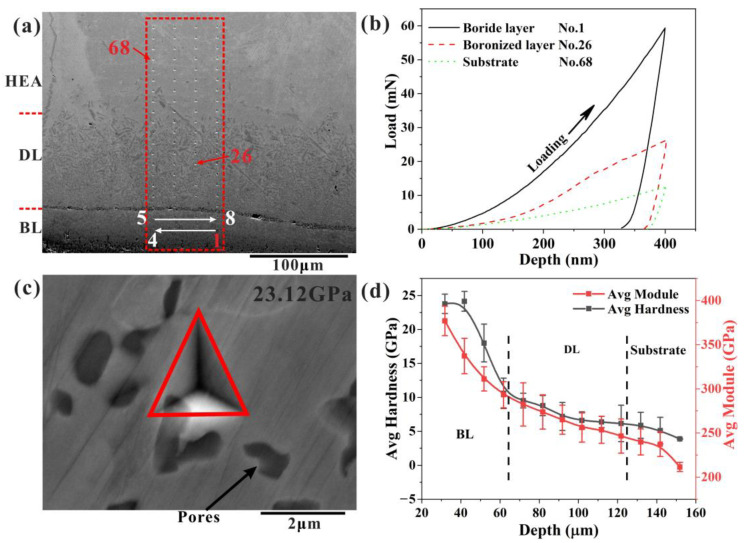
(**a**) Measurement position of the nanoindentation test, (**b**) load-displacement curves of the three representative nanoindentation points (Nos.1, 26, 68); (**c**) the indentation morphology on the single-phase layer; (**d**) average modulus and hardness vs. distance from the surface.

The effect of boronizing on the mechanical properties of CoCrFeNiMn alloys was investigated. The cross-sectional morphology of the boronized CoCrFeNiMn alloys after the nanoindentation test is shown in Figure 9a. 80 locations were tested according to the S-shaped path, and a series of load-displacement curves were obtained, as shown in Figure 9b. For the same indentation depth (400 nm), the 68th indentation band requires the lowest load, the 1st indentation point requires the highest load, and the 26th indentation point load is in between. The indentation morphology on the single-phase BL is shown in c. The microhardness and Young’s modulus results at different locations were obtained, as shown in Table 3. It is indicated that the hardness of the BL is significantly higher than the DL and substrate, while the surface hardness of the HEA was markedly improved after boronizing. The variation curve of indentation hardness with depth was obtained from Table 3, as shown in Figure 9d. The average hardness of the substrate is 3.9 ± 0.1 GPa, and the average hardness of the BL is 23.8 ± 1.4 GPa, while the trend of modulus and hardness is similar. After the boriding treatment, a multilayer reinforced layer consisting of numerous high-hardness borides is formed on the surface of HEA, resulting in a significant improvement in the surface hardness and modulus of HEA.

**Figure 10:**
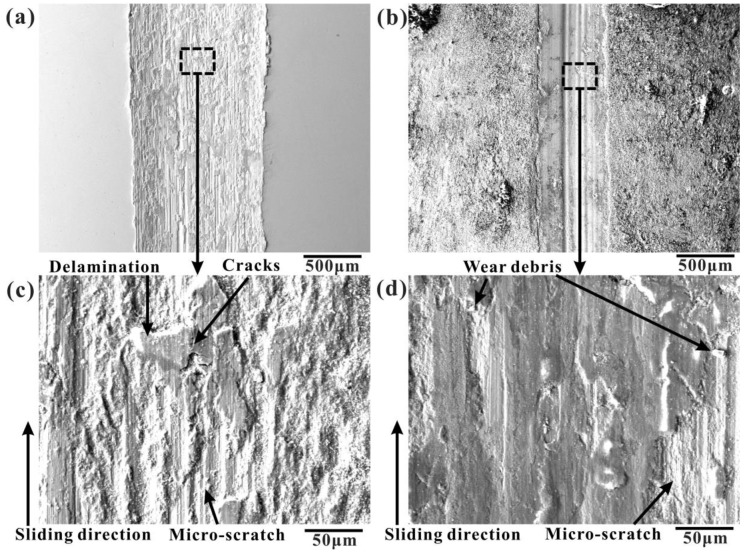
SEM images of the worn surface of the CoCrFeNiMn HEA: (**a**,**c**) as-cast CoCrFeNiMn HEA; (**b**,**d**) CoCrFeNiMn HEA boronized at 950 °C for 9 h.

The tribological behavior of the strengthening layer in dry conditions was investigated using reciprocating ball-on-plate tests. The wear tracks of unboronized and boronized alloys at 950 °C for 9 h under dry conditions were investigated using SEM, as shown in Figure 10. The width of friction tracks on the surface of the boronized CoCrFeNiMn HEA was significantly reduced, and the interior of the wear tracks was smoother compared with the unboronized samples, as shown in Figure 10a,b. The periodically localized fracture of the surface layer and the periodic accumulation and elimination of debris on the worn surface of CoCrFeNiMn HEA were observed, as shown in Figure 10c. The plastic deformation along the groove, some lamellar delamination, and pits could be seen. In addition, it is also found that the surface is attached to some wear debris, indicating that it is adhesive wear. The boronized HEA has no peeling pits, and more parallel grooves emerge. However, the small abrasive dust is significantly reduced, and a large number of parallel grooves are observed. The occurrence of parallel furrows is a representative characteristic of abrasive wear, as shown in Figure 10d.

**Figure 11:**
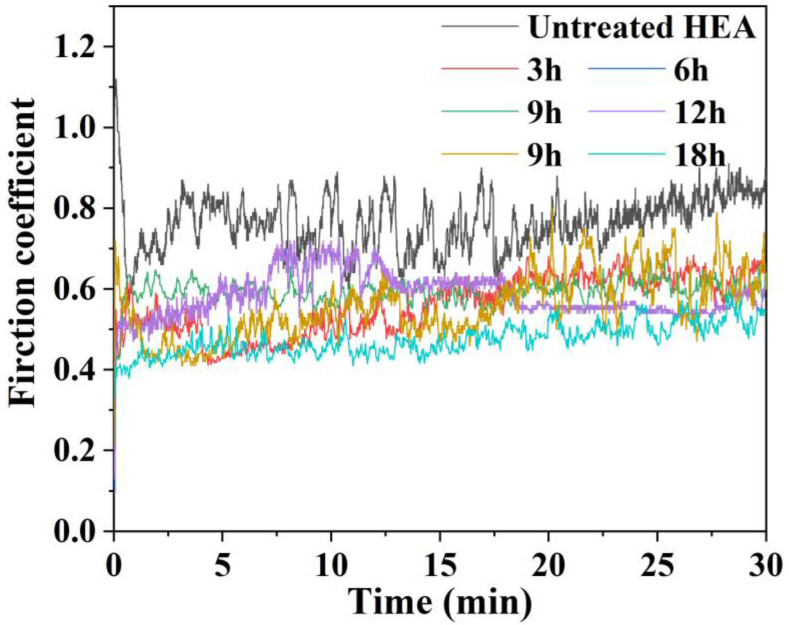
Friction coefficient plots of unboronized and boronized samples.

Figure 11 shows the friction coefficient curves of the unboronized and boronized HEA. The friction coefficient of the boronized alloy (0.48~0.61) is lower than that of the unboronized alloy (0.86), and the friction coefficient shows a decreasing trend. Among these boronized samples, the friction coefficient of the 18h sample was the lowest at 0.48, while some broad waves with relatively large fluctuations are observed in the friction coefficient curve of the untreated alloy. Boronizing can effectively improve the wear resistance of HEA surfaces due to their higher surface hardness and larger thickness of the strengthened layer.

## 4. Conclusions

In this work, a reinforced layer on the surface of HEA was successfully synthesized by powder-pack boriding. The microstructure, microhardness, wear resistance, and boriding mechanism were investigated. The following conclusions can be summarized:

(1) The microstructure of the surface layer is mainly composed of MB-type boride and M_2_B-type boride. The original interface of the surface layer is located at the interface between the BL and the diffusion layer, where element B diffuses inward and metal elements diffuse outward.
(2) The activation energy and frequency factor of the B element in CoCrFeMnNi HEA are 206.93 kJ/mol and 9.15 × 10^−5^ m^2^/s, respectively. Increasing the boronizing duration and temperature resulted in an increase in the BL and diffusion layer thickness.
(3) The surface strengthening of CoCrFeMnNi HEA was achieved by the boriding treatment. Its surface microhardness has significantly increased from 3.9 ± 0.1 Gpa to 23.8 ± 1.4 Gpa. The surface layer shows a lower friction coefficient of 0.48 than that of the substrate (0.86). Depending on the wear mechanism, adhesive wear mainly occurs in unboronized samples, and abrasive wear is the main wear mechanism in boronized samples.

The findings of this study could provide insights into designing and developing high-hardness and high-wear resistance alloys through surface treatment, which can be used in the cast aluminum industry and allow HEA to be applied in special environments and increase service life.

## Figures and Tables

**Table 1:** Values of the frequency factor and the activation energy.

	Frequency Factor D_0_, m^2^/s	Activation Energy Q, kJ/mol
Boronized HEA	9.15 × 10^−5^	206.93

**Table 2:** EPMA elemental analysis of the boronized samples for 9h.

Position	Compositions						Phase
	B	Cr	Mn	Fe	Co	Ni	
1	45.5	13.5	11.4	13.6	11.2	4.8	Ni-poor
2	30.8	5.0	13.7	10.7	13.8	26.0	Ni-rich and Cr-poor M_2_B-type boride
3	31.9	18.2	11.9	17.0	14.9	6.1	Ni-poor M_2_B-type boride
4	29.8	28.8	12.2	12.5	10.2	6.5	Cr-rich and Ni-poor M_2_B-type boride
5	1.9	9.4	21.4	24.0	23.5	19.8	Cr-poor FCC
6	27.9	32.3	11.9	9.8	9.4	8.7	Cr-rich and Ni-poor M_2_B-type boride
7	1.6	20.5	20.5	21.9	19.4	17.1	Substrate

**Table 3:** Average modulus and hardness of the boronized sample for 18 h.

Group	Distance from the Surface (µm)	Serial Number	Group Avg Modulus (GPa)	Group Avg Hardness (GPa)
1	31.82	1–4	377.0 ± 16.6	23.8 ± 1.4
2	41.82	5–8	337.1 ± 20.0	24.1 ± 1.5
3	51.82	9–12	311.2 ± 13.7	18.0 ± 2.8
4	61.82	13–16	293.4 ± 14.9	10.6 ± 2.2
5	71.82	17–20	282.3 ± 24.5	9.5 ± 1.1
6	81.82	21–24	273.8 ± 19.5	8.8 ± 1.5
7	91.82	25–28	265.0 ± 16.4	7.2 ± 2.1
8	101.82	29–32	256.2 ± 16.6	6.6 ± 1.3
9	111.82	33–36	253.5 ± 15.2	6.4 ± 0.8
10	121.82	37–40	246.5 ± 19.3	6.2 ± 2.7
11	131.82	41–44	239.8 ± 14.8	5.9 ± 1.9
12	141.82	45–48	237.3 ± 14.0	5.1 ± 2.0
13	151.82	49–80	211.5 ± 5.2	3.9 ± 0.1

## Data Availability

Data will be made available on request.
